# Evidence of validity, reliability, and measurement invariance of the psychological characteristics related to sport performance questionnaire in Colombian athletes

**DOI:** 10.3389/fpsyg.2026.1860055

**Published:** 2026-09-28

**Authors:** Jaime Arenas-Granada, Deysi Torres-Penagos, Andrés Colorado, Erica Cano, Haney Aguirre-Loaiza, Elena Hernández-Hernández

**Affiliations:** 1 Faculty of Physical Education, Recreation and Sport, Politécnico Colombiano Jaime Isaza Cadavid, Medellín, Antioquia, Colombia; 2 Department of Sports and Informatics, Universidad Pablo de Olavide, Sevilla, Spain; 3 Unilasallista Corporación Universitaria, Caldas, Antioquia, Colombia; 4 Department of Psychology, Universidad Católica de Pereira, Pereira, Risaralda, Colombia; 5 Grupo de Investigación SEJ-570, Sevilla, Spain

**Keywords:** measurement invariance, psychological characteristics, reliability, sport performance, sport psychology, validity

## Abstract

**Background:**

The assessment of psychological characteristics is crucial for the intervention and monitoring of sports performance. However, there is a lack of instruments for measuring psychological characteristics in Colombian athletes.

**Methods:**

This research aimed to examine the validity and reliability of the scores obtained from the Psychological Characteristics related to Sport Performance Questionnaire (CPRD in Spanish). Two complementary studies were conducted: Study 1 included 1.305 athletes, comprising 412 women and 893 men, with an age range between 13 and 59 years, divided into age groups; Study 2 included 478 athletes, comprising 150 women and 328 men, with an age range between 13 and 37 years.

**Results:**

The four-factor, 37-item CPRD demonstrated acceptable internal consistency for stress control (SC), influence of performance evaluation (IPE), and motivation (M), but inconsistent reliability for team cohesion (TC) (ω = 0.64; α = 0.65), although overall reliability was satisfactory. The four-factor CPRD showed adequate model fit indices. Although the questionnaire demonstrated adequate concurrent validity, convergent validity was not satisfactory. Inverse correlations were observed between the four-factor CPRD and burnout dimensions, and acceptable ratios supporting discriminant validity were identified, along with measurement invariance across sport modality, sex, and age groups (ΔCFI and/or ΔRMSEA ≤ 0.01).

**Conclusion:**

The four-factor CPRD provides partial evidence of validity and reliability for assessing relevant psychological variables in the Colombian sports context; however, interpretations based on factor-level scores should be made with caution.

## 1 Introduction

Decision-making in the sports field, both applied and research-based, is grounded in rigorous assessment processes, which are fundamental for the diagnosis and psychological intervention of athletes (Dosil, 2008; Gümüşdag and Ilhan, 2022; Tenenbaum et al., 2012; Weinberg and Gould, 2018). To ensure their effectiveness, assessment instruments must provide solid evidence of validity and reliability (Anastasi and Urbina, 1998). In the Latin American context, one of the instruments used is the Psychological Characteristics Related to Sports Performance (CPRD by Spanish acronym) questionnaire. The CPRD was proposed by Gimeno et al. (2001), who provided the first psychometric evidence based on internal structure, reliability, and scoring in Spanish athletes. The CPRD questionnaire originated from the linguistic adaptation from English to Spanish of the Psychological Skills Inventory for Sport (PSIS) (Mahoney et al., 1987; Mahoney, 1989). This suggests that the CPRD questionnaire may have inherited negative heuristics (Lakatos, 1983) from the PSIS that threaten the fit of the original model (Gimeno et al., 2001), both in terms of measurement and theory.

In the absence of a theoretical framework for both the PSIS and the CPRD, a conceptual model for the CPRD was proposed two decades ago (Gimeno et al., 2001; Gimeno and Buceta, 2010). The purpose was to account for the interaction between psychological skills and athletic behavior. This initiative considered five factors: (1) *Stress control*: related to responses to the demands of training and competition and to potentially stressful situations requiring control. (2) *Influence of performance evaluation*, involving characteristics of the athlete's response to situations and contexts in which they evaluate their own performance or are evaluated by significant others; (3) *Motivation*, which encompasses the athlete's interest in excelling day by day through the effort to achieve their set goals; (4) *Mental ability*, including the psychological skills that influence athletic performance, specifically regarding goal setting, performance analysis, and cognitive self-regulation; and (5) *Team cohesion*, which refers to the athlete's integration within their team, including the interpersonal relationships the athlete has with teammates, the attitude and level of satisfaction the athlete feels within the group, and the importance they place on team spirit (Gimeno et al., 2001).

In Latin America, the CPRD questionnaire has had a significant impact on sports psychology research specifically, in Latin American observational studies (Aguirre-Loaiza et al., 2020; Arias et al., 2016; Barbosa-Granados et al., 2024; Hernández et al., 2021; Lorenzón et al., 2022; Quintero-Ovalle et al., 2023; Tamez and Verástegui, 2022; Zapata-Huenullán et al., 2023) as well as in Spanish studies (Fradejas and Espada-Mateos, 2018; Gimeno et al., 2007; Gomez-Marcos and Sanchez-Sanchez, 2019; Hevilla-Merino and Castillo-Rodríguez, 2018; Mahamud et al., 2005; Olmedilla et al., 2015, 2017, 2018, 2019b; Vélez-Alcazar et al., 2026; Olmedilla et al., 2021a). Furthermore, the CPRD has been used in research with experimental designs (Moreno-Fernández et al., 2019; Olmedilla et al., 2010, 2019a, 2021b). This body of studies appears to indicate the instrument's relevance and potential practical implications, given that the CPRD questionnaire has often been used as a tool to assess and intervene in the psychological skills of elite athletes (Gimeno and Buceta, 2010; Nieto and Olmedilla, 2001; Olmedilla et al., 2010; Roffé, 2009). In particular, the questionnaire has proven useful for assessing the psychological skills of promising athletes (Dosil, 2001; Ramírez-Muñoz and Prieto-Andreu, 2021), which are a distinguishing factor in the identification and selection of athletic talent.

Validation and adaptation studies of the CPRD show varying results depending on the country and the population assessed. In Spain, the original instrument exhibited a five-factor structure with 55 items, explaining 63% of the variance, with adequate reliability across three factors, while motivation and mental ability showed lower coefficients (Gimeno et al., 2001). Subsequently, the CPRD-EF for soccer coaches was analyzed using principal component analysis, identifying a single component that integrated the questionnaire's five factors, with high factor loadings, particularly in stress control and motivation, confirming a global psychological construct. Reliability showed adequate values (α = 0.76), with no items affecting internal consistency; furthermore, normative scales were developed for Andalusian coaches (Mendoza-Pérez et al., 2006). Subsequently, specific adaptations confirmed this structural variability, such as the CPRD-F for soccer players, which reduced the instrument to 40 items across five factors, with high overall internal consistency (α = 0.85) and 42.11% explained variance (López-López et al., 2013), and the CPRD-M for mountain sports, which proposed a 45-item, four-factor model with excellent overall reliability (α = 0.90) and satisfactory factor fit (Ramos et al., 2018). In Argentina, the adaptation resulted in an even shorter version with 25 items and four factors, which showed good factor fit (CFI = 0.92; NNFI = 0.91; RMSEA = 0.06) and adequate to very good internal consistency, supporting its use in team sports (Pagano and Vizioli, 2020). Finally, in Colombia, the CPRD retained adequate overall reliability estimated via Bayesian analysis (ω = 0.87; α = 0.87) and allowed for the construction of normative scales, although the results indicated the need for caution in interpreting the mental skill and team cohesion factors due to their lower internal consistency (Álvarez et al., 2025).

Based on the above and in accordance with the review conducted, the CPRD questionnaire is frequently used in research and constitutes a useful tool in the field of sport psychology; however, there are no known studies specific to the Colombian context that have empirically evaluated the validity and reliability of the CPRD questionnaire. Given this situation and the lack of interest in psychometric research in sports psychology, it is essential to develop studies that bridge this gap and contribute to knowledge regarding the measurement and assessment of psychological abilities. Consequently, the objectives of this study were: First, to review the evidence of factor validity and evaluate factor models of the CPRD; based on the selected model, the second objective was to review the evidence of the discriminant, convergent, concurrent, and reliability (internal consistency) of the CPRD questionnaire scores.

## 2 Materials and methods

### 2.1 Study 1—factor analysis and reliability of the CPRD

#### 2.1.1 Participants

Through a purposive sampling design, *n* = 1,305 athletes participated, comprising 412 women (31.6%) and 893 men (68.4%), with an age range of 13 to 59 years (*M* = 18.66, SD = 5.38; Women: *M* = 17.96, SD = 4.82; Men: *M* = 18.99, SD = 5.59). In terms of sport type, team sports predominated, with 78.62% of participants (*n* = 1,026), compared to 21.38% who practiced individual sports (*n* = 279), and age groups, with 50.65% of 13–17 years (*n* = 661), and with 49.35% of 18–59 years (*n* = 644). With regard to competitive classification, the largest proportion was in the league category (*n* = 629; 48.20%), followed by high performance (*n* = 478; 36.63%), and collegiate (*n* = 198; 15.17%).

#### 2.1.2 Techniques and instruments

##### 2.1.2.1 *Ad hoc* self-report

A self-report consisting of two parts was provided: (1) *sociodemographic information* (age, sex, educational level, socioeconomic status); (2) *sports information* (type of sport, competitive classification, sports experience).

##### 2.1.2.2 Questionnaire on psychological characteristics related to sports performance

The CPRD questionnaire was administered, consisting of 55 items and five factors: Stress Control (SC = 20 items), Influence of Performance Evaluation (IPE = 12 items), Motivation (*M* = 8 items), Mental Skills (MSK = 9 items), and Team Cohesion (TC = 8 items). It uses a five-point response format that assesses the degree of agreement using a Likert-type attitude scale: (0 = *strongly disagree*, 1 = *disagree*, 2 = *neutral*, 3 = *agree*, 4 = *strongly agree*). The questionnaire has proven useful with athletes aged 13 and older (Gimeno et al., 2001). Furthermore, the questionnaire demonstrated reliability through internal consistency: (SC: α = 0.88; IPE: α = 0.72; M: α = 0.67; MSK: α = 0.34; TC: α = 0.78). It also provided evidence of its internal structure through factor validity, with 63% of variance explained by five factors (Gimeno et al., 2001).

#### 2.1.3 Procedure

In both studies, non-probability sampling was implemented based on participants' willingness to take part in the research. Likewise, data were collected from March 2023 to November 2025 by visiting various leagues and sports clubs in municipalities of the Aburrá Valley (Medellín, Envigado, Sabaneta) and the municipality of Guarne in eastern Antioquia, as well as the Coffee Region (Pereira, Armenia). The guidelines of the American Educational Research Association, American Psychological Association, and National Council on Measurement in Education (2018) regarding standards for educational and psychological testing were followed. Data collection from the athletes proceeded (with prior authorization from the leaders and coaches of the sports leagues and clubs, following informed consent from each participant or their legal representative). The athletes were evaluated before training. Training sessions were conducted with those responsible for data collection to ensure they understood the nature of the statements (items) in the CPRD questionnaire and the athlete exhaustion scale, which allowed for appropriate feedback to athletes who might have difficulty understanding the questions during the evaluation. The collected data are protected by personal data protection provisions Ley (1581, 2012).

#### 2.1.4 Ethical considerations

National and international ethical guidelines were followed. To ensure best practices in the use of psychometric materials, the provisions of Chapter VI, Articles 45 through 48 Ley (1090, 2006) were implemented. Likewise, Colombian scientific, technical, and administrative standards for health research [Ministry of Health, Resolución 8430 (1993)] and the Universal Declaration of Ethical Principles for Psychologists (International Union of Psychological Science, 2008) were applied. Participants provided informed consent and assent. Study 1 was approved by the Ethics Committee of Pablo de Olavide University, Seville, Spain (Code 25/1-26, 11/03/2025).

#### 2.1.5 Statistics and data analysis

Statistical analysis was performed using JASP software version 0.19.3 (JASP Team, 2025). An exploratory data analysis was performed to identify missing and outlier values and make corrections in accordance with technical recommendations (Hair et al., 2014). Descriptive statistics such as absolute frequency, mean, standard deviation, and interquartile range were calculated for the purpose of conducting a descriptive analysis of sociodemographic and sports-related variables. Furthermore, Mardia's coefficient (Mardia, 1970) was used to test the assumption of multivariate normality; the value obtained for multivariate kurtosis was 3,775.54, with a significant *p*-value of 0.01, which is above the threshold of 70. Consequently, the Weighted Least Squares with Mean and Variance (WLSMV) robust estimator was selected due to the ordinal nature of the data. Subsequently, a confirmatory factor analysis (CFA) was conducted to evaluate the adjustment of the first four-order (multidimensional) models. The fit was assessed using the χ^2^ test, where χ^2^ = chi-square statistic; *df* = degrees of freedom; CFI = Comparative Fit Index; TLI = Tucker–Lewis Index; SRMR = standardized root mean square residual; RMSEA = root mean square error of approximation; CI = confidence interval, using conventional criteria for good fit: CFI and TLI greater than 0.90, and RMSEA and SRMR values less than 0.08 (Hu and Bentler, 1999). Likewise, standardized factor loadings (λ) equal to or greater than 0.35 were considered adequate (Johnson and Stevens, 2001). Reliability based on internal consistency was estimated using Cronbach's alpha (α) and McDonald's omega (ω) (Raykov and Hancock, 2005). Subsequently, the invariance of measurement was analyzed with the CFA multigroup method considering groups defined by sport modality, sex, and age groups. The difference in CFI (ΔCFI) or RMSEA (ΔRMSEA) was used to determine whether models had more or fewer constraints, with configural invariance as the base model and progressing to metric, strong, and strict invariance. To determine an adequately constrained and appropriate model at each stage of invariance, the ΔCFI or ΔRMSEA had to present values <0.01 (Byrne, 2008; Chen, 2007).

## 3 Results

### 3.1 Factor structure of the CPRD questionnaire

Confirmatory factor analysis was conducted to estimate four confirmatory factor models, using as a baseline the CPRD model proposed by Gimeno et al. (2001), the four-factor model with 41 items, the four-factor model with 37 items, and a short three-factor version with 9 items. The original model did not yield satisfactory values for the Comparative Fit Index (CFI = 0.873) and the Total Likelihood Index (TLI = 0.867), whereas the four-factor and three-factor models yielded satisfactory fit indices.

According to the fit indices reported in Table 1, a progressive improvement in model fit is observed as the number of factors and items is reduced. The initial five-factor model (55 items) showed acceptable fit, with a χ^2^ (1,420) value of 7,384.94, CFI = 0.873, TLI = 0.867, SRMR = 0.068, and RMSEA = 0.057, 90% CI [0.055, 0.058], although the incremental indices fell below the recommended criterion of 0.90. Subsequently, the four-factor model with 41 items showed a substantial improvement in fit, χ^2^ (813) = 3,041.00, CFI = 0.938, TLI = 0.934, SRMR = 0.057, and RMSEA = 0.046, 90% CI [0.044, 0.044], achieving adequate values on the comparative indices. This trend was reinforced in the four-factor model with 37 items, which showed a satisfactory fit, χ^2^(623) = 2,234.36, CFI = 0.949, TLI = 0.946, SRMR = 0.057, and RMSEA = 0.045, 90% CI [0.043, 0.047] (Figure 1). Finally, the three-factor model with 9 items presented the best overall performance, with χ^2^ (24) = 80.29, CFI = 0.969, TLI = 0.954, SRMR = 0.040, and RMSEA = 0.042, 90% CI [0.032, 0.053], comfortably meeting conventional criteria for good fit, suggesting a more parsimonious and empirically robust factor structure.

**Table 1:** Fit indices of the evaluated factor models (*N* = 1305).

Model	χ^2^	df	CFI	TLI	SRMR	RMSEA [90% CI]
Five factors (55 items)	7,384.94	1420	0.873	0.867	0.068	0.057 [0.055,0.058]
Four factors (41 items)	3,041.00	813	0.938	0.934	0.057	0.046 [0.044,0.044]
**Four factors (37 items)**	**2,234.36**	**623**	**0.949**	**0.946**	**0.057**	**0.045 [0.043,0.047]**
Three factors (9 items)	80.29	24	0.969	0.954	0.040	0.042 [0.032,0.053]

CFI, comparative fit index; TLI, tucker–lewis index; SRMR, standardized root mean square residual; RMSEA, root mean square error of approximation; CI, sterval. Bold values indicate the selected model: the four-factor (37-item) solution, which showed the best overall fit (χ2 = 2,234.36, df = 623, CFI = 0.949, TLI = 0.946, SRMR = 0.057, RMSEA [90% CI] = 0.045 [0.043, 0.047]).

**Figure 1:**
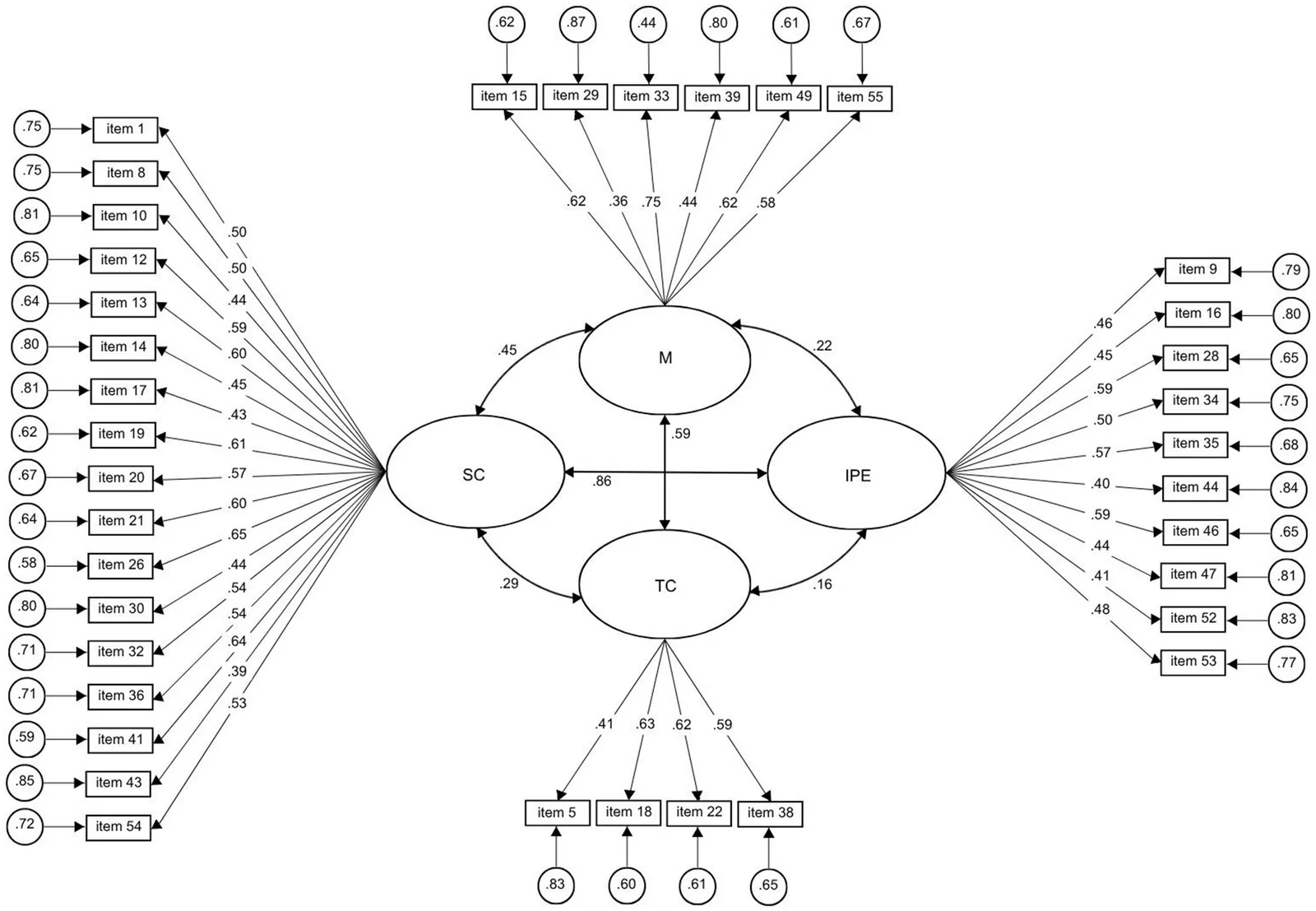
Four-factor model correlated with 37 items of the CPRD.

### 3.2 Internal consistency reliability

The results varied in terms of the internal consistency coefficients of the factors across the different models evaluated (see Table 2). In the original five-factor model (55 items), the SC, IPE, and M dimensions showed adequate levels of reliability, with values of ω = 0.86 and α = 0.86 for SC, ω = 0.76 and α = 0.76 for IPE, and ω = 0.71 and α = 0.73 for M; in contrast, the factors MSK (ω = 0.56; α = 0.36) and TC (ω = 0.45; α = 0.53) had coefficients below the recommended threshold. In the four-factor model with 41 items, adequate coefficients were maintained for SC (ω = 0.85; α = 0.86), IPE (ω = 0.76; α = 0.77), and M (ω = 0.63; α = 0.62), while the TC dimension reached moderate values (ω = 0.58; α = 0.60), leading to the removal of MSK from the model. In contrast, the **four-factor** model with **37 items** was more balanced, as SC (ω = 0.87; α = 0.87), IPE (ω = 0.75; α = 0.76), and M (ω = 0.71; α = 0.73) reached acceptable levels, while TC showed lower reliability (ω = 0.64; α = 0.65). Meanwhile, the three-factor model with 9 items showed a general reduction in internal consistency, with values of ω = 0.62 and α = 0.62 for SC, ω = 0.53 and α = 0.54 for IPE, and ω = 0.42 and α = 0.39 for M. Finally, all four models indicated values above 0.70 overall.

**Table 2:** Reliability by model, factor, and overall.

Model	SC		IPE		M		MSK		TC		CPRD	
	ω	α	ω	α	ω	α	ω	α	ω	α	ω	α
Five factors (55 items)	0.86	0.86	0.76	0.76	0.71	0.73	0.56	0.36	0.45	0.53	0.87	0.89
Four factors (41 items)	0.85	0.86	0.76	0.77	0.63	0.62	—	—	0.58	0.60	0.89	0.89
**Four factors (37 items)**	**0.87**	**0.87**	**0.75**	**0.76**	**0.71**	**0.73**	**—**	**—**	**0.64**	**0.65**	**0.90**	**0.90**
Three factors (9 items)	0.62	0.62	0.53	0.54	0.42	0.39	—	—	—	—	0.71	0.69

ω = McDonald's Omega coefficient; α = Cronbach's alpha. The dash indicates that the factor was not included in the model. The selected factor is shown in bold. MSK, Mental skills.

### 3.3 Selected factor model—four factors (37 items)

The 37-item, four-factor model was selected based on the following global evaluation criteria: (1) moderate values for the CFI, TLI, SRMR, and RMSEA fit indices; (2) the narrowest RMSEA confidence interval; and the following localized evaluation criteria: (3) acceptable (≥ 0.70) and/or marginal (≥ 0.60) factor reliability coefficients; and (4) item factor loadings ≥ 0.30.

The retention of four factors was supported by their applied relevance, interpretability, and adequate internal consistency for CE, IER, and M. Although CHE showed marginal reliability (ω = 0.66; α = 0.65), it was provisionally retained to preserve its specific content related to team cohesion. In contrast, Mental Skills was excluded because it encompasses heterogeneous psychological strategies and, as acknowledged by Gimeno et al. (2001), may not represent “a single and comprehensive construct,” which is consistent with its low internal consistency in the original study (α = 0.34). It should also be noted that the CPRD has an applied conceptual foundation but does not operationalize a formal psychological theory or a unified model.

The 37-item, four-factor model was selected because it provided the best balance between parsimony and content validity. Although the nine-item model showed slightly better fit, its excessive brevity reduced both the representation of the conceptual domain and factor reliability. Therefore, a simpler model is not necessarily more valid; parsimony should be pursued without removing essential components of the construct.

The removal of the MSK factor was justified by its poor psychometric performance in the five-factor, 55-item solution, as evidenced by poor internal consistency (ω = 0.56; α = 0.36), weak and predominantly negative factor loadings—Item 2 (λ = −0.11), Item 7 (λ = −0.32), Item 23 (λ = 0.28), Item 25 (λ = −0.51), Item 37 (λ = −0.31), Item 40 (λ = −0.51), Item 45 (λ = −0.49), Item 48 (λ = −0.37), and Item 50 (λ = −0.58) and a low proportion of explained variance (*R*^2^ = 0.17). Moreover, this finding is consistent with the poor internal consistency of this factor reported in previous psychometric studies of the CPRD (Álvarez et al., 2025; Gimeno et al., 2001; Mendoza-Pérez et al., 2006), supporting the removal of the MSK domain to achieve a more consistent and interpretable factorial structure. Furthermore, given the absence of a formal psychological theory underpinning the CPRD, it was not possible to establish theory-based criteria for assessing the relevance of this domain.

According to Table 3, the SC dimension had mean factor loadings of λ =0.53 (range =0.39–0.65) and a mean R^2^ of.29, indicating moderate representation of the items in the factor. For IPE, the factor loadings were lower (mean λ = 0.49; range = 0.49–0.59), with an average explained variance of *R*^2^ = 0.24. Meanwhile, the M and TC dimensions showed the highest loadings (λ = 0.56 in both cases), along with average R^2^ values of.33, reflecting an adequate and consistent contribution of the items to the four latent factors. Figure 1 shows the covariances between factors, loadings, and measurement errors for each item.

**Table 3:** Summary of standardized factor loadings (λ) and explained variance (*R*^2^) by factor.

Factor	No. of items	Mean λ	Min–Max λ	Mean R^2^	*R^2^* min–max
SC	17	0.53	0.39 −0.65	0.29	0.15 −0.42
IPE	10	0.49	0.49 −0.59	0.24	0.16 −0.35
M	6	0.56	0.36 −0.75	0.33	0.13 −0.56
TC	4	0.56	0.41 −0.63	0.33	0.17 −0.40

SC, stress control; IPE, influence of performance evaluation; M, motivation; TCC, team cohesion.

## 4 Materials and methods

### 4.1 Study 2—discriminant, convergent, and concurrent validity of the CPRD questionnaire

#### 4.1.1 Participants

A total of (*N* = 478) Colombian athletes, comprising 150 women (31.4%) and 328 men (68.6%), with an age range of 13 to 37 years (*M* = 16.74, *SD* = 4.56; Women: *M* = 18.61, *SD* = 5.28; Men: *M* = 15.88, *SD* = 3.91). With regard to educational level, lower secondary education was the most common (*n* = 217; 45.40%), followed by upper secondary (*n* = 161; 33.68%) and undergraduate (n = 61; 12.76%) education; technical (*n* = 23; 4.81%) and vocational secondary (*n* = 13; 2.72%) education, and graduate education (*n* = 1; 0.21%) were less common, with 0.42% of missing values (*n* = 2). With reference to socioeconomic status, the largest proportion corresponded to the lower-middle (*n* = 186; 38.91%), followed by the middle (*n* = 127; 26.57%) and lower (*n* = 74; 15.48%) strata, while the upper-middle (*n* = 47; 9.83%), lower-middle (*n* = 26; 5.44%), and high (*n* = 10; 2.09%) strata showed lower frequencies; 1.76% of missing values were identified (*n* = 8). In terms of the type of sport, most participants practiced team sports (*n* = 428; 89.54%), compared to 10.46% who practiced individual sports (*n* = 50). Finally, in the competitive classification, 43.93% were classified as high performance (*n* = 210), 35.36% as performance (*n* = 169), and 20.71% as federated athletes (*n* = 99).

#### 4.1.2 Instruments

##### 4.1.2.1 Questionnaire on psychological characteristics related to sports performance

The four-factor CPRD questionnaire was administered, which included: Stress Control (SC = 17 items); Influence of Performance Evaluation (IPE = 10 items); Motivation (M = 6 items), and Team Cohesion (TC = 4 items). The response format is consistent with the five-factor CPRD from Study 1. It uses a five-point Likert-type attitude scale to assess the degree of agreement: (0 = *strongly disagree*, 1 = *disagree*, 2 = *neutral*, 3 = *agree*, 4 = *strongly agree*). The questionnaire has proven useful with athletes aged 13 and older (Gimeno et al., 2001). The internal consistency reliability values were satisfactory and were estimated in the results of Study 1, specifically in Table 2.

##### 4.1.2.2 Athlete burnout questionnaire

The Athlete Burnout Questionnaire (ABQ) was administered, which contains 15 items and three factors: Emotional/Physical Exhaustion (EPE = 5 items), Sport Devaluation; (SDE = 5 items), and Reduced Sense of Accomplishment (RSA = 5 items). It uses a five-point response format that assesses frequency via a Likert-type attitude scale: (1 = *almost never*, 2 = *rarely*, 3 = *sometimes*, 4 = *often*, 5 = *almost always)*. All items are formulated such that the higher the score on the items, the greater the burnout identified, except for items 1 and 14, which measure the opposite (Raedeke and Smith, 2001). Furthermore, the questionnaire demonstrated good reliability through the temporal stability of the scores: (EPE: *R* = 0.92; RSA: *R* = 0.86; SDE: *R* = 0.92) (Raedeke and Smith, 2001).

#### 4.1.3 Ethical considerations

For the second study, the ethical provisions of Study 1 were retained. This work was approved by the Ethics Committee of the Politécnico Colombiano Jaime Isaza Cadavid, Medellín, Colombia (Code 20610801, 11/24/2022).

#### 4.1.4 Statistics and data analysis

As in Study 1, the analysis plan was carried out using JASP software version 0.19.3 (JASP Team, 2025). In response to the second objective, this analysis used Spearman's correlation (*r*_*s*_) between the four-factor CPRD and the burnout factors to generate evidence of discriminant validity, and correlations with years of sports experience to generate evidence of concurrent validity. Convergent validity was examined using the Average Variance Extracted (AVE). The AVE is defined as the mean value of the squared factor loadings of the indicators associated with the construct (i.e., the sum of the squared factor loadings divided by the number of indicators). The minimum acceptable value for AVE is 0.50. Additionally, the Heterotrait–Monotrait ratio (HTMT) was also used to determine discriminant validity. The HTMT ratio is obtained by comparing the correlation between different dimensions with the correlations within the same construct. An HTMT value below.85 indicates the presence of discriminant validity.

## 5 Results

### 5.1 Discriminant and concurrent validity

According to Table 4, the reduction in the sense of accomplishment showed moderate negative correlations with SC (*rs* = −0.33), IPE (*rs* = −0.31), and M (*rs* = −0.37), and a low negative correlation with TC (*rs* = −0.22), indicating that higher levels in these dimensions are associated with a lower perception of ineffectiveness; Similarly, physical and emotional exhaustion showed low negative correlations with SC (*rs* = −0.20), IPE (*rs* = −0.17), M (*rs* = −0.18), and TC (*rs* = −0.18). Sports devaluation also showed negative associations, ranging from low to moderate in magnitude, with SC (*rs* = −0.24), IPE (*rs* = −0.24), M (*rs* = −0.32), and TC (*rs* = −0.19). On the other hand, years of experience correlated positively with SC (*rs* = 0.30), IPE (*rs* = 0.20), M (*rs* = 0.19), and TC (*rs* = 0.12), showing relationships of low to moderate magnitude. The HTMT ratio results indicated adequate evidence of discriminant validity among the model's dimensions. All HTMT coefficients were below the conservative cutoff of 0.85, with values ranging from 0.14 to 0.83. Regarding convergent validity, the average variance extracted values ranged from 0.24 to 0.32 (SC = 0.30; IPE = 0.24; M = 0.31; TC = 0.32). None of the factors reached the recommended cutoff of 0.50, indicating that the factors explain between 24% and 32% of the variance in their respective indicators.

**Table 4:** Descriptive statistics and correlations for discriminant and concurrent validity.

Validation measures	Descriptive statistics			Correlations			
	M	SD	Range	SC	IPE	M	TC
CPRD Questionnaire							
SC	2.71	0.81	0–4				
IPE	2.36	0.80	0–4	0.63^***^			
M	3.41	0.69	1–4	0.42^***^	0.25^***^		
TC	3.61	0.66	0–4	0.27^***^	0.17^***^	0.31^***^	
Validity							
Discriminant							
RSA	1.89	0.87	1–5	−0.33^***^	−0.31^***^	−0.37^***^	−0.22^***^
EPE	1.64	0.72	1–5	−0.20^***^	−0.17^***^	−0.18^***^	−0.18^***^
SDE	1.68	0.79	1–5	−0.24^***^	−0.24^***^	−0.32^***^	−0.19^***^
HTMT				0.83			
				0.39	0.19		
				0.27	0.14	0.57	
Convergent							
AVE				0.30	0.24	0.31	0.32
Concurrent							
Years of experience	7.87	4.17	1–29	0.30^***^	0.20^***^	0.19^***^	0.12^*^

RSA, reduced sense of accomplishment; EPE, emotional/physical exhaustion; SDE, sport devaluation; HTMT, heterotrait–monotrait; AVE, average variance extracted; SC, stress control; IPE, influence of performance evaluation; M, motivation; TC, team cohesion. ^*^p < 0.05, ^**^p < 0.01, ^***^p < 0.001.

The findings from the multigroup confirmatory factor analysis supported configural, metric, scalar, and strict measurement invariance of the CPRD across sport modality, sex, and age groups. Across successive models, the absolute changes in CFI ranged from.001 to.009, while changes in RMSEA ranged from.000 to.004. Because all values remained below the recommended threshold of.010, the factor structure, loadings, thresholds/intercepts, and residuals can be considered equivalent across the examined groups (Table 5).

**Table 5:** Invariance of measurement by CFA multigroup for CPRD.

Grouping variable	Invariance (Model)	χ^2^	df	CFI	ΔCFI	RMSEA	ΔRMSEA
Sport modality	1. Configure	2.858.62	1,246	0.948		0.045	
	2. Metric	3.192.46	1,279	0.939	0.009	0.048	0.003
	3. Strong	3.286.92	1,312	0.937	0.002	0.048	0.000
	4. Strict	3.425.62	1,349	0.933	0.004	0.049	0.001
Sex	1. Configure	2.778.11	1,246	0.947		0.043	
	2. Metric	3.097.92	1,279	0.938	0.009	0.047	0.004
	3. Strong	3.161.03	1,312	0.937	0.001	0.047	0.000
	4. Strict	3.260.27	1,349	0.934	0.003	0.047	0.000
Age groups	1. Configure	2.654.88	1,246	0.956		0.042	
	2. Metric	2.813.73	1,279	0.952	0.004	0.043	0.001
	3. Strong	2.914.19	1,312	0.950	0.002	0.043	0.000
	4. Strict	3.050.25	1,349	0.947	0.003	0.044	0.001

X^2^ (df) = chi-square statistic (χ^2^) with degrees of freedom (df), CFI, comparative fit index; TLI, tucker-lewis index; SRMR, standardized root mean square residual; RMSEA, root mean square error of approximation; Δ = difference.

## 6 Discussion

The four factorial solutions showed progressively better fit as the number of items was reduced. The 55-item solution exhibited the least satisfactory fit (CFI = 0.873, TLI = 0.867, SRMR = 0.068, and RMSEA = 0.057), whereas the 41- and 37-item solutions yielded adequate and highly similar values. Although the nine-item model showed slightly better CFI, TLI, and RMSEA values, these differences were small; the only appreciable difference was observed in the SRMR, which was lower for the nine-item model (0.040) than for the 37-item model (0.057). Nevertheless, compared with the other three solutions, the 37-item factorial solution demonstrated satisfactory reliability across its dimensions, supporting its selection as a balanced solution in terms of overall fit, internal consistency, and representation of the construct's content. Accordingly, the nine-item model may be considered the most parsimonious alternative for screening purposes, rapid assessments, or studies seeking to reduce respondents' cognitive burden, provided that its three factors retain adequate reliability and sufficiently represent the construct's content. However, the 37-item model may be more useful when the aim is to conduct a broader and more differentiated assessment of athletes' psychological characteristics. The literature on the development of short forms recommends that item reduction preserve conceptual coverage, internal consistency, and relationships with external variables, rather than being based exclusively on improved factorial fit (Sexton et al., 2014). Furthermore, solutions derived from numerous sample-specific decisions should be replicated, because data-driven modifications may capitalize on chance associations and exhibit limited stability in new samples (MacCallum et al., 1992). The CPRD also demonstrated strict measurement invariance across sport modalities, sex, and age groups. This finding indicates that the scale's factorial structure, factor loadings, intercepts, and measurement errors are equivalent across the groups compared (Leitgöb et al., 2023). This conclusion is supported by the absolute values of ΔCFI ≤ 0.01 and/or ΔRMSEA < 0.015, which are widely accepted criteria for establishing measurement invariance in psychometric studies, even when multiple groups are examined (Chen, 2007). Finally, strict invariance is rarely achieved in psychometric research; however, when established, it provides the highest degree of confidence for making group comparisons because even measurement errors are equivalent across groups (Byrne, 2008; Chen, 2007; Leitgöb et al., 2023). The CPRD's fulfillment of these criteria suggests the absence of measurement bias attributable to sport-related and sociodemographic factors, such as sport modality, sex, or developmental maturation. This finding is particularly relevant given that psychological characteristics may be influenced by sport-related and developmental factors.

Likewise, the reliability of the overall questionnaire reached adequate values (α = 0.88; ω = 0.90), indicating solid internal consistency of the instrument as a whole. In the Colombian context, instrumental studies of the CPRD remain scarce, although various observational studies have reported evidence of reliability based on internal consistency (Aguirre-Loaiza et al., 2020; Arias et al., 2016; Barbosa-Granados et al., 2024; Hernández et al., 2021; Quintero-Ovalle et al., 2023). For example, Barbosa-Granados et al. (2024) reported a reliability coefficient of ω = 0.87 in athletes with disabilities, while Quintero-Ovalle et al. (2023) found high estimates in Colombian athletes in individual and team sports (α = 0.88; ω = 0.90). Similarly, Aguirre-Loaiza et al. (2020) reported values of ω = 0.89 in soccer referees, and Hernández et al. (2021) reported coefficients of ω = 0.88 in athletes participating in the Paranational Games. Taken together, these findings suggest a relatively stable consistency of the CPRD's global scale across different sports populations, supporting its applicability in various contexts of Colombian sports. With regard to the instrument's factors, Quintero-Ovalle et al. (2023) indicated that they all exhibit adequate levels of internal consistency, with estimates above 0.70 (SC α = 0.86, IPE α = 0.72, M α = 0.77, MSK α = 0.75, and TC α = 0.73). However, the elimination of the MSK factor does not merely represent statistical refinement; it also entails a reduction in the conceptual domain assessed by the original CPRD. Specifically, the assessment of strategies related to goal setting, performance analysis, visualization, and cognitive self-regulation is lost. Nevertheless, the heterogeneity of these contents, their low internal consistency since the original study (Gimeno et al., 2001), and their poor performance in the present study (ω = 0.56; α = 0.36; weak factor loadings; *R*^2^ = 0.17) indicate that they do not constitute an empirically well-defined domain and that the factor lacks both logical and empirical adequacy (Bacharach, 1989). Therefore, the 37-item solution retains the assessment of four relevant CPRD domains (SC, IPE, M, and TC), because they have demonstrated satisfactory internal consistency (Álvarez et al., 2025; Gimeno et al., 2001; Jaenes-Sánchez et al., 2010; Mendoza-Pérez et al., 2006; Zapata-Huenullán et al., 2023) and the ability to discriminate between groups (Aguirre-Loaiza et al., 2020; Arias et al., 2016; Barbosa-Granados et al., 2024; Gomez-Marcos and Sanchez-Sanchez, 2019; Hernández et al., 2021; Jaenes-Sánchez et al., 2010; Olmedilla et al., 2019b, 2021a; Vélez-Alcazar et al., 2026). However, it should not be considered conceptually equivalent to the original five-factor instrument, but rather a reduced version with a more restricted scope. This modification limits direct comparisons with previous studies that used either the total score or the MSK subscale. Comparisons should therefore be restricted to the four retained factors while accounting for potential differences in their item composition. Consequently, the absence of the MSK factor should be acknowledged as a limitation of content coverage, and the proposed structure requires independent replication and additional validity evidence before it can be regarded as a substitute for the original CPRD.

In this regard, reports on internal consistency using different factor structures of the CPRD have estimated reliability values. Spanish mountaineers were assessed using a tetra-factorial model (Ramos et al., 2018), and the internal consistency estimates were above 0.70 (TC: α = 0.82, IPE: α = 0.79, Self-confidence: α = 0.82, Concentration: α = 0.74). Similarly, a tetra-factorial model reported by Pagano and Vizioli (2020) in Argentine athletes is consistent with these values (SC: α = 0.84, ω = 0.87; Self-confidence: α = 0.81, ω = 0.85; TC: α = 0.89, ω = 0.89; M: α = 0.70, ω = 0.80). It is important to note that the comparison of these confirmatory models with the original (penta-factorial) model by Gimeno et al. (2001) has been approached with caution, as their results do not guarantee the conceptual equivalence and reproducibility of the latent structure of the original model. As reported in the CPRD factor-revision studies (Pagano and Vizioli, 2020; Ramos et al., 2018) and due to statistical modeling, some items have been removed and the meaning of others has been modified, altering the CPRD factors.

With regard to the CPRD factors, the values appear to vary, both across factors and across the reviewed studies. Our results highlighted that reliability was satisfactory for SC (ω = 0.86; α = 0.85), IPE (ω = 0.76; α = 0.76), and M (ω = 0.73; α = 0.73), with the exception of TC, which indicated inconsistency in the scores (ω = 0.66; α = 0.65). The original CPRD study with Spanish athletes (Gimeno et al., 2001) reported consistent values for TC (α = 0.88), IPE (α =0.72), and TC (α =0.78), while the dimensions of M (α = 0.67) and MSK (α = 0.34) reported low estimates. Along the same lines, the report on consistency coefficients in athletes with disabilities (Hernández et al., 2021) indicates similar results: TC ω = 0.83; IPE ω = 0.77; M ω = 0.61; MSK ω = 0.44; TC ω = 0.40. The study by López-López et al. (2013) reported adequate reliability values for self-confidence (α = 0.77), stress and anxiety control (α = 0.75) and motivation (α = 0.67); whereas the dimensions of attitude and mental preparation (α = 0.59) and concentration (α = 0.68) did not achieve satisfactory values. Conversely, the literature shows inconsistencies regarding the structural stability of the original model (Gimeno et al., 2001), given that while some studies modified the names of the factors (López-López et al., 2013; Pagano and Vizioli, 2020; Ramos et al., 2018), others fully retained the initial latent structure (Álvarez et al., 2025; Mendoza-Pérez et al., 2006). In this regard, Brown (2015) indicates that the factors are justified based on theory.

On the other hand, the findings corroborating the discriminant validity of the four-factor CPRD were supported by significant negative correlations with the dimensions of sports burnout. In particular, the associations with reduced sense of accomplishment ranged from −0.22 to −0.37, with physical and emotional exhaustion ranging from −0.17 to −0.20, and with sports devaluation ranging from −0.19 to −0.32. The low to moderate magnitude of these relationships, along with their theoretically consistent direction, suggests that the psychological skills assessed by the CPRD are empirically distinct from burnout syndrome, although they maintain the expected inverse relationship. Taken together, these results provide adequate evidence of discriminant validity. The concurrent validity of the CPRD was supported by positive and significant correlations between its dimensions and years of athletic experience. The association was moderate for stress control (rs = 0.30, *p* < 0.001) and low for the influence of performance evaluation (rs = 0.20), motivation (rs = 0.19), and team cohesion (*r* = 0.12). The AVE values (0.24–0.32) were below the conventional criterion of.50, indicating limited convergence among the indicators of each factor. This finding may be related to the conceptual breadth and content heterogeneity of the CPRD dimensions, as well as to the complexity of certain item formulations inherited from the original PSIS. Although the so-called “negative heuristics” may reduce factor loadings by introducing wording-related method effects, the study design does not allow them to be identified as the primary cause of the low AVE values. In applied settings, the limited proportion of common variance may reduce score precision and the instrument's ability to differentiate between athletes with similar levels of the attribute. Consequently, the CPRD should be used as an orienting and complementary measure rather than as a stand-alone diagnostic instrument, and its results should be integrated with other sources of psychological information.

These results suggest that athletes with longer careers exhibit higher levels of psychological skills, which is consistent with the expertise or deliberate practice hypothesis in sport (Ericsson et al., 1993). Although the effect sizes were small to moderate, they are to be expected in field studies and support the instrument's ability to reflect differences related to competitive experience. The results obtained are consistent with recent international evidence. Like the study by Garrido-Muñoz et al. (2024), positive and significant correlations were observed between age and years of practice with certain psychological variables; however, the effect sizes were small, indicating a limited effect. Furthermore, the findings partially contrast with those reported by Quintero-Ovalle et al. (2023), who found no significant associations. Overall, the evidence suggests that, although sports experience may contribute to the development of psychological skills, its influence is not decisive, and other factors, such as structured psychological training or sport-specific demands, may play a more significant role.

### 6.1 Limitations and future research directions

Several limitations should be considered for future studies. First, the sampling design was not random-representative, and therefore does not allow for the generalization of the results, which may introduce selection bias, despite a large and heterogeneous sample of athletes from different regions and the underrepresentation of men in the sample. Second, the CPRD was administered in both paper-and-pencil and online formats, which may vary and introduce social desirability bias. Third, we were unable to obtain evidence of test–retest reliability, which would have allowed us to incorporate another type of reliability evidence.

Therefore, future research may address some of these limitations. For example, exploring the psychometric properties of the four-factor CPRD under a different theoretical and methodological framework, specifically incorporating the *multidimensional graded response model* within the framework of item response theory; this will allow for the explicit modeling of multidimensionality, appropriate handling of ordinal data, estimation of item-invariant parameters, precise assessment of measurement error, and advanced analysis of item functioning (Muñiz, 1997; Samejima, 1969). Finally, the absence of four-factor CPRD norms in Colombia with a technical basis of validity and reliability highlights the need to develop context-specific norms differentiated by sex, age group, and sport modality, which would provide coaches and sports psychologists with greater flexibility in evaluating athletes.

## 7 Conclusion

The evidence obtained supports the usefulness of the four-factor CPRD as an instrument with partial evidence of validity and reliability for assessing relevant psychological variables in the Colombian sports context. From an applied perspective, the questionnaire constitutes a potentially useful tool for psychological assessment processes and for the design and monitoring of interventions aimed at optimizing athletic performance. In particular, the Colombian version of the four-factor CPRD allows for interpretations supported by preliminary psychometric evidence; however, these interpretations should be made with caution, especially at the factor level. Consequently, the instrument may serve as a supportive resource for sport psychologists in the assessment and planning of intervention programs, as well as for coaches in understanding and monitoring psychological factors associated with performance. Thus, its use may contribute to informed decision-making in both research and professional practice. Nevertheless, future studies should continue to accumulate evidence of validity and reliability to strengthen the interpretation and use of its scores.

## Data Availability

The original contributions presented in the study are included in the article/supplementary material, further inquiries can be directed to the corresponding author.
